# Comparing the Effectiveness of Multicomponent Sleep-Promoting Interventions on the Sleep Quality of Menopausal Women: A Quasi-Experimental Study

**DOI:** 10.3390/healthcare10030559

**Published:** 2022-03-16

**Authors:** Mei-Hsiang Lin, Ping-Ru Hsiao, Hsiu-Chin Hsu

**Affiliations:** 1 School of Nursing, National Taipei University of Nursing and Health Sciences, Taipei 112303, Taiwan; mhlin5452@gmail.com; 2 Department of Nursing, Chang Gung University of Science and Technology, Taoyuan 333424, Taiwan; pjhsiao@mail.cgust.edu.tw; 3 Department of Nursing, Linkou Chang Gung Memorial Hospital, Taoyuan 333423, Taiwan; 4 Graduate Institute of Health Care, Chang Gung University of Science and Technology, Taoyuan 333424, Taiwan; 5 Department of Internal Medicine, Chang Gung Memorial Hospital, Taoyuan 333423, Taiwan

**Keywords:** menopause, sleep wake disorders, progressive muscle relaxation, sleep hygiene

## Abstract

Sleep disturbance is considered one of the hallmarks of the common symptoms experienced by women during and after menopause. This study aimed to compare the effectiveness of two different multiple-component, sleep-promoting interventions on the sleep quality of menopausal women. A quasi-experimental study and repeated measured design, with a four-week sleep-promoting intervention, was conducted. A total of 123 eligible participants were recruited from a health center in northern Taiwan and divided into the progressive muscle relaxation plus sleep hygiene (PMRS), the meditative movement relaxation plus sleep hygiene (MMRS), or control group at a 1:1:1 ratio. The Chinese version of Pittsburgh sleep quality index and actigraphy were used to assess the sleep disturbances of menopausal women. The subjective sleep data was collected before, immediately after the intervention, 8 weeks, and 12 weeks after the intervention. The results showed that the global score of subjective sleep quality and its components were significantly improved after both interventions. Additionally, the MMRS was superior to the PMRS for subjective sleep quality. Moreover, the objective sleep indices indicated that sleep latency was reduced after both the interventions. These findings can serve as a reference for nurses when caring for menopausal women with sleep disturbance.

## 1. Introduction

Sleep disturbance is considered one of the hallmarks of the common symptoms experienced by women during and after menopause; it is also one of the primary reasons for women to seek medical help during this period [1,2]. Prairie et al. [1] conducted a survey of 625 women, aged 40–60, to investigate their reasons for visiting general gynecologic clinics or specialized menopause clinics, and they discovered that up to 80% of the participants had sleep-related complaints. Additionally, numerous studies have showed that the prevalence of sleep disturbance varied from 16% to 42% in premenopausal women, 39% to 47% in perimenopausal women, and 35% to 60% in postmenopausal women, respectively [3,4]. Long-term sleep deprivation can have negative physical, as well as psychological, impacts, markedly lowering the quality of life (QoL) of menopausal women [5].

To date, many women are now turning to non-pharmacological treatments, including exercise and complementary and alternative medicine, such as meditation, exercise, and yoga, to alleviate menopausal symptoms [3,6]. Notably, when considering the appropriate strategies to ameliorate the sleep disturbances during menopause, the physical, mental, and social changes women experience should be accounted for. On the basis of factors affecting the sleep quality of menopausal women, studies on the improving the sleep quality of menopausal women have mostly emphasized stress relaxation by applying meditative movements relaxation (MMR), progressive muscular relaxation (PMR), diaphragmatic breathing, and sleep hygiene as an intervention for sleep disturbance [7,8,9]. A MMR that combines specific body movements (or positions), meditation, and controlled breathing to promote both physical and mental harmony has been widely adopted for usage in ameliorating sleep problems [10]. Most studies on the effectiveness of MMR for improving sleep disturbances have indicated that it can significantly improve sleep quality, without adverse side effects. Patients who practiced MMR have demonstrated increased sleep duration and reported feeling more refreshed after waking up [11,12]. To sum up the above statement, low-intensity aerobic body movements, emptying one’s mind, regulated breathing, and concentrating can help alleviate physical discomfort, reduce stress, and promote sleep quality. PMR was originally developed by Jacobsen in 1938, in which the body and mind are greatly relieved from any tension and anxiety [13]. PMR involves taking several deep breaths, followed by progressively tensing and relaxing (for 15 and 30 s, respectively) nine muscle groups from the head to the lower limbs [14]. The primary mechanism of PMR in relieving stress and sleep disturbances lies in the conscious activation of the parasympathetic nervous system, both during and after PMR [15]. It has also been suggested that PMR is a systematic technique that can be used to achieve deep somatic restfulness, together with parasympathetic dominance, which reduces anxiety, a consequence in improved sleep quality [16,17].

Several empirical studies have attested to the positive effects of PMR on promoting sleep quality [18,19]. Zarbakhsh et al. [19] carried out the RCT to examine the efficacy of progressive muscular relaxation on sleeping quality among clinical nurses. The results demonstrated that PMR could significantly improve nurses’ sleep quality. In contrast, a randomized controlled trial conducted by Blanaru et al. [17], who investigated individuals with posttraumatic stress disorder, implementing the PMR and music intervention to improve sleep quality. The result showed that there were no significant differences in sleep parameters (objective and subjective sleep) after the PMR intervention; however, the findings showed that following PMR intervention, sleep latency was reduced from 124 to 29 min in the experimental group [17]. Moreover, sleep hygiene comprises of maintaining regular sleep wake times, limited alcohol, caffeine, and nicotine use prior to bed time, and sleep environments conducive to sleep has been widely applied in various ethnic groups for sleep health promotion [20]. Everitt et al. [21] pointed out that, although sleep hygiene is often used to solve sleep disturbance, due to its low cost and easy availability, most general practitioners concluded sleep hygiene to be insufficient to address the sleep problem. In conclusion, sleep hygiene is an intervention that guides people with sleep disturbances to more respectable, adequate, and lifestyle-oriented practices to improve sleep quality, but it often fails, due to the ill-discipline in maintaining good practice [22]. Based above statements, sleep hygiene could be as an auxiliary strategy to improve sleep quality in menopausal women. 

Several studies have also noted that multiple-component interventions, integrating two or more techniques, can often achieve favorable results for sleep-related complaints [6,23]; to the best of our knowledge, there are limited studies to address which of the components had the greatest effect on improving sleep quality for menopausal women. Moreover, most of the above-mentioned sleep related information comes from the subjective perception of participants. Using a combination of both subjective statements and objective sleep monitors to examine sleep condition was rare. This study aimed to compare the effectiveness of two different multiple-component sleep-promoting interventions, namely progressive muscle relaxation plus sleep hygiene (PMRS) and meditative movement relaxation plus sleep hygiene (MMRS) on the sleep quality of menopausal women. The primary hypothesis was that the MMRS would more effectively improve sleep quality of menopausal women than PMRS.

## 2. Materials and Methods

### 2.1. Design

A quasi-experimental study and repeated measured design, with a four-week sleep-promoting intervention, was conducted to assess the sleep quality of menopausal women. 

### 2.2. Participants and Sampling

Convenience sampling was used and participants were recruited from a health center in northern Taiwan based in a district with a population greater than 120,000. Participants were included in this study if aged 45–55 years old, diagnosed with menopause by obstetrician, they have a Pittsburgh sleep quality index (PSQI) score greater than 5, and they have no mental illness or severe medical conditions, such as cancer or cardiopulmonary diseases. The exclusion criteria were habitual user of hormonal products and participation in an ongoing sleep promotion course during the past six months. The sample size was estimated with G-Power 3.1.9, and data of the three groups were tested using the repeated-measures between-factors ANOVA, assuming α = 0.05, power = 0.8, a Cohen’s medium effect size of 0.25, and a medium level autocorrelation of 0.5 for interactions 50% of the time. Four times of measurements were performed for participants across three groups, and the required sample size was estimated to be 102, with an actual power of 0.81. However, considering that the attrition rate would be existed in the longitudinal study, the number of recruited participants should be 20% more than the actual number. Therefore, the total number of recruited participants was set at 123. Eligible participants were divided into three groups (MMRS, PMRS, and control group) at a 1:1:1 ratio. 

**Figure 1:**
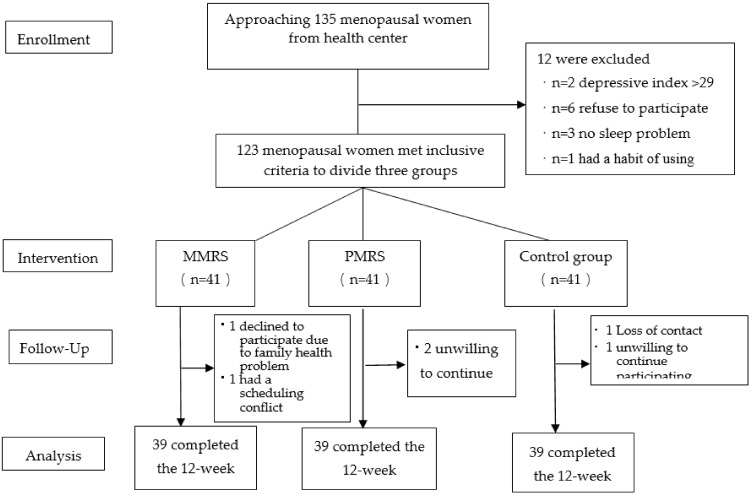
The flowchart of study enrollment, participation, intervention, and analysis inclusion.

In this study, a total of 135 menopausal women were invited to participate, of whom 12 refused or did not meet the inclusion criteria. The remaining 123 were divided into three groups. During the intervention, two women in each group withdrew their participation for personal reasons (e.g., being sick or having to tend to a sick family member) or time constraints, leaving 117 participants (39 per group) to complete the intervention (Figure 1). 

### 2.3. Instruments

#### 2.3.1. Demographic Data

The demographic data including age, marital status, educational level, economic status, and the presence of chronic disease, social activity, sleep environment, and retirement were collected. 

#### 2.3.2. The Chinese Version of Pittsburgh Sleep Quality Index (CPSQI)

The CPSQI, which was originally developed by Buysse et al. and translated and standardized by Tsai et al. [24], contains 19 questions divided into seven major components: sleep latency, subjective sleep quality, sleep duration, sleep efficiency, sleep disturbances, use of sleeping medication, and daytime dysfunction. Each component was equally weighted, using 0 to 3 points, and the total score of all seven components ranged from 0 to 21. “Poor sleep” has been defined as a CPSQI score greater than 5. Regarding the reliability of the CPSQI, it showed an internal consistency and split-half reliability of 0.82–0.83 and 0.94, respectively [24]. In the present study, the Cronbach’s α was 0.77. In addition, five sleep disorder experts, including two women’s health nursing professors, one physician of sleep clinical, one gynecological nurse specialist, and one experiencing menopausal woman, were invited to evaluate the clarity, feasibility, and suitability of the CPSQI. The CVI of the CPSQI in present study was 0.90. Additionally, five menopausal women with an elementary school level of education were recruited to read the items included in the questionnaire prior to the review. Individual interviews were conducted with the five women, regarding their understanding of the questionnaire content, to evaluate the clarity of the sentences and meanings and create validity for the data collection.

#### 2.3.3. Actigraphy

The Actiwatch-64, produced by the Mini Mitter Company, OR, USA (2005), was employed to objectively assess the sleep parameters of menopausal women in this study. The indices include sleep latency (SL) is the time (minutes) taken to fall asleep after turning out the lights; time in bed (TIB) is the time (minutes or hours) from turning out the lights to getting out of bed at the end of sleep period; total sleep time (TST) is the time (minutes or hours) of TIB spent asleep; sleep efficiency is TST divided by TIB, as a percent; wake after sleep onset (WASO) is the time awake after falling asleep divided by TST, as a percent; snooze is time (minutes) took to get out of bed after waking up. The Actiwatch is a wristwatch-sized device that has been widely used in clinical practice and researched to investigate sleeping problems. The validity of the Actiwatch has been confirmed by using the polysomnography to distinguish between being asleep and awake [8]. Concurrently, the reliability of the Actiwatch exhibited a correlation coefficient of 0.88 with polysomnography.

#### 2.3.4. Intervention

A sleep-promoting intervention, which emphasized stress relaxation and lasted for four weeks, with one 2-h session per week, was conducted. In the first session, both the MMRS and PMRS groups were instructed on issues relating to the sleep problems of menopausal women. In addition, both groups were instructed on topics relating to sleep hygiene, and they were given a brochure to help them integrate the lessons into their daily lives. Additionally, at the end of every session, the researcher would allow time for discussion, during which the participants were encouraged to ask questions and share their experiences, and the researcher would also emphasize the importance of daily practice and log keeping.

Meditative movement relaxation relied on a simple eight-move stress-relieving exercise, integrating the principles of qiqong, exercise, and meditation with muscle stretching, special moves, as well as diaphragmatic breathing skills, to achieve relaxation and then improve sleep. The participants were required to immediately practice the demonstrated techniques. Upon entering a relaxed state, the participants were instructed to sit still and allow their minds to settle, while focusing on breathing control, with the aim of alleviating anxiety and finding mental calm. The eight-move exercise was taught by the principal investigator (PI), who was accompanied by two experts that provided on-site assistance, direction, and consultation. The PI and one of the research assistants had more than one year of experience practicing the exercise, and the other assistant was a qiqong practitioner with 10 years teaching experience (see Table 1). 

Progressive muscle relaxation focused on the practice of PMR to improve sleep disorder. In each session, the participants were divided into groups, containing 9–10 members per group, the researcher explained and demonstrated the procedures of the PMR, and then the participants were required to immediately repeat them. The whole session lasted for 90 min. At the end of the session, CDs with PMR instructions were issued to the participants, and they were instructed to spend 30 min every night lying down to gradually relax and fall asleep to the instructions on the CD. Participants in the control group received no intervention. At the end of the intervention, they were given the same sleep-promoting brochure that was given to the experimental groups (see Table 1).

### 2.4. Ethical Consideration

This study was approved by the Ethical Committee of the Chang Gung Medical Foundation Institutional Review Board with code of IRB99-3190B. The purposes of this study and the participants’ rights, as well as obligations, were clearly explained to the participants by the researchers. Participants were fully informed that they were free to leave this study at any time if they felt uncomfortable, and all participants signed informed consent forms before participating in the study.

### 2.5. Data Collection

All participants were required to complete the questionnaires, including demographic information, and PSQI, and they were also required to wear an Actiwatch sleep monitor to establish their baseline data before the intervention. To avoid possible discrepancies between the data taken on weekdays and those on weekends, the Actiwatch was only worn from Monday to Thursday. Instructions for wearing the Actiwatch were delivered orally, and the participants were also provided with an instruction sheet. Because sleep parameter on the first night wearing the Actiwatch may be underestimated, participants wore the watch for three consecutive nights. Additionally, to prevent the biasing Rosenthal effect that occurs when participants’ responses are affected by the researchers’ expectation, one of research assistants, who was blind to participants’ assigned intervention, was responsible for collecting data. The participants were informed that their subjective sleep-related data (minus demographic information) would be assessed again immediately after the intervention, 8 weeks, and 12 weeks after the intervention, in order to analyze the immediately and delay effects. The participants were also asked to wear the Actiwatch twice, before and after the intervention.

### 2.6. Data Analyses 

The PASW Statistics 26.0 (IBM Inc., Armonk, NY, USA) for Windows was used for data analyses. A *t* test for independent samples, chi-squared test, and Fisher’s exact test were employed to examine the homogeneity between groups, based on their demographic characteristics and the baseline data derived from the sleep quality. The significant differences were entered as covariates in the further analyses. The generalized estimating equation was used to test the effects of the intervention on sleep quality. A *p* value of 0.05 was considered statistically significant.

## 3. Results

The basic characteristics of the three groups of women were summarized in Table 2. The baseline characteristics among the three groups were not significantly different, except for the levels of education. Additionally, there were no significant differences in the scores of PSQI and actigraphic sleep parameter, among the three groups, except for the percentage of waking time. 

In terms of the mean global sleep quality score, the results showed that all the participants were classified with poor sleep quality, but the overall scores of the experimental groups revealed a gradual improvement. The mean global sleep quality scores for the pretest, posttest, 8-weeks, and 12-weeks follow-up tests were 11.41, 8.56, 8.41, and 7.41, respectively, for the PMRS group, and 10.95, 7.28, 6.41, and 6.67, respectively, for the MMRS group (Table 3). In addition, the four measurements of the participants’ subjective sleep quality, sleep latency, sleep duration, habitual sleep efficiency, sleep disturbance, use of sleeping pills, and daytime dysfunction revealed improvement, as well, in both the PMRS and MMRS groups. By contrast, the scores of the four measurements in the control group were fluctuating between good and poor (Table 3).

According to the scores obtained from actigraphic indicators, the two measurements of the participants’ sleep latency, percentage of time awake, snooze time, total sleep time, and sleep efficiency presented improvement, as well in both the PMRS and MMRS groups. The results showed that the mean score for percentage of time awake decreased from 13.75 to 10.01 in the PMRS group and from 12.29 to 9.72, respectively, in the MMRS group. Conversely, in the control group, the ratio percentage of time awake increased from 12.42 (pretest) to 14.05 (posttest), showing an upward growth (Table 4).

After controlling the level of education and employment status, the score of the global PSQI showed that there was a significant difference in the interaction between the PMRS and the T2, T3, and T4 (Wald *x*^2^ = 14.03, *p* < 0.01; Wald *x*^2^ = 5.61, *p* < 0.05; Wald *x*^2^ = 20.72, *p* < 0.01) (Table 5). Similarly, there was a significant difference in the interaction between the MMRS and T2, T3, and T4 (Wald *x*^2^ = 33.32, *p* < 0.01; Wald *x*^2^ = 25.14, *p* < 0.01; Wald *x*^2^ = 58.50, *p* < 0.01). Furthermore, when the PMRS was set as the control group, there was a significant difference in the interaction between the two groups at three follow-up times (Wald *x*^2^ = 4.40, *p* < 0.05; Wald *x*^2^ = 6.75, *p* < 0.01; Wald *x*^2^ = 7.11, *p* < 0.01), and the average PSQI scores of the MMRS in the T2, T3, and T4 were 1.46, 1.85, and 1.92 points better, respectively, than the PMRS group (Table 5). This indicates that the MMRS was more effective than the PMRS in improving the subjective sleep quality (Table 5). 

In terms of the actigraphic sleep parameter, both the PMRS and MMRS attained a significant difference in their interactions, with T2 in sleep latency, after controlling the level of education and employment status (Wald *x*^2^ = 4.36, *p* = 0.04; Wald *x*^2^ = 6.05, *p* = 0.01). This indicates that both the PMRS and MMRS were effective at improving sleep latency. For the percentage of nocturnal awaking, there was a significant difference in the interaction between the MMRS and T2 (Wald *x*^2^ = 5.55, *p* = 0.02). This indicates that the MMRS was effective at lowering the percentage of “awakened time.” Moreover, in the total sleep time, there was a significant difference in the interaction between the PMRS and T2 (Wald *x*^2^ = 5.21, *p* = 0.02). This demonstrates that the PMRS was effective in improving the total sleep time (Table 5).

However, when the PMRS was set as the control group, there was no significant difference in the interaction between the two groups at two measurements, nor did the sleep latency, total sleep time, time awake during sleep, or snooze time (Table 5).

## 4. Discussion

The primary aim of the present study was to compare the effectiveness of two different multiple-component, sleep-promoting interventions on the sleep quality of menopausal women. The results of this study echoed the hypothesis, in which the MMRS would more effectively improve sleep quality of menopausal women than PMRS. Both MMRS and PMRS were effective for reducing the subjective sleep disturbances and decreasing sleep onset latency on actigraphic indicators. Only the MMRS was effective for reducing wake time, yet the PMRS was effective for increasing the total sleep time.

With regard to the participants’ subjective sleep quality, both the PMRS and MMRS groups exhibited significant differences immediately post-intervention, as well as after 8 and 12 weeks. Our findings are in accordance with those of numerous studies that have noted the effectiveness of stress reduction interventions on sleep quality [2,6,25,26]. Some evidence-base studies confirmed that the efficacy of CBT would be effective on pre-sleep cognitive arousal, rumination, and worry, consequently improving sleep [2,26]. Additionally, Guthrie et al. [27] synthesized a number of empirical studies regarding the effect of promoting sleep quality and concluded that CBT, as a first-line treatment in healthy midlife women with insomnia, was recommended. From this point of view, menopausal women with sleep disturbances who might experience maladaptive thinking, somatic hyperarousal, and stress dysregulation, due to hormonal changes during this transition, would likely benefit from MMRS and PMRS. Nevertheless, we further compared the effectiveness of the PMRS and MMRS on subjective sleep quality, and our results exhibited that the overall mean score of PSQI in the MMRS was lower than that of PMRS. It indicated that the effectiveness of the MMRS was superior to the PMRS in improving the subjective sleep quality for menopausal women. A possible explanation for the effectiveness of the MMRS in improving subjective sleep quality was the synergistic effect created between the combination of physical movement and psychological approaches to foster states of relaxation, counteract intrusive thoughts, decrease body tension, and the integration of sleep hygiene in daily life. Moreover, the results of this study were similar with the findings of a randomized controlled trial, conducted by Ong et al. [6], who evaluated the effect of mindfulness meditation on sleep quality among community-dwelling adults with chronic insomnia. Ong et al. [6] noted a significant drop in the total nocturnal awake time and increase in the total sleep hours. Ong et al. further stated that, because insomnia has a high prevalence among women who are also more willing to receive alternative nonpharmacological treatment, MMRS might be an ideal option for women who are undergoing a critical physio-psychological transition by using meditation to improve their sleep. Studies have also revealed that medium-to-low-intensity exercise can significantly improve subjective sleep quality and insomnia [25,26,28]. Our results are congruent with those findings. The participants in the MMRS group were taught a locally developed, medium-to-low-intensity exercise, which was coupled with meditation and deep breathing to lower symptom-related stress and promoted sleep quality, through activating the parasympathetic nervous system, thus decreasing alarm reactions [29].

The subjective sleep quality of the participants in the PMRS group was also significantly different after the intervention in the present study. Our findings are consistent with those of numerous studies that support the effectiveness of PMR in promoting sleep quality, particularly in terms of sleep latency and total sleep duration, because practicing contracting and relaxing muscles can reduce muscle tension, nervousness, and anxiety, thus improving sleep [17,18]. Another reason that the subjective sleep quality attained significance in the present study could be the personality traits of the participants, who were community-dwelling menopausal women that voluntarily sought cancer screening. Consequently, they were proactive, attached importance to personal health, and were relatively willing to accept and practice sleep-promoting interventions. The majority had no previous experience of receiving similar interventions, and most were extremely interested in health-promotion topics. Although all aspects of the participants’ subjective sleep quality indicated significant improvement, care should be taken to determine whether this was the result of a novelty effect, which could lower the external validity of the findings because this was the first time that most of the participants had received such an intervention.

Notably, the findings of this study show that the global PSQI scores were still greater than 5 in both experimental groups after intervention. A possible explanation is that the dose and duration of interventions may play a crucial role at this point. More time devoted to practice stress reduction techniques would presumably result in more favorable outcomes [30]. Compared with some effective interventions that have been conducted for 6 months, our intervention program was relatively short, being conducted 30 min per day for 12 weeks. In this circumstance, although the PSQI scores exhibited gradual improvement during the intervention, the participants were unlikely to achieve the standard required for good quality of sleep. Concerning the objective sleep parameters obtained from the Actigraphic, the results revealed that sleep latency exhibited a significant improvement in both the MMRS and PMRS groups. By contrast, the percentage of nocturnal awakening revealed a significant improvement in only the MMRS group. The results of this study showed that the percentage of total awake time reported by participants in the MMRS group changed from 12.3% to 9.7% in the pretest and follow-up tests, respectively. Conversely, the control group reported 16.4% and 18.1% in the pretest and follow-up test, respectively. These findings indicate that women with menopause should be encouraged to practice the combined techniques of meditation and body movement relaxation when experiencing abnormal nocturnal awakening frequency. 

There were some notable strengths in this study, including dividing the participants by their places of residence to the experimental and control groups, as well as using two methods to measure their sleep (i.e., verifying the self-reported survey answers with data measured by actigraphic devices). 

### Limitations

There were also several limitations in this study. Despite being continually reminded at the end of each teaching session to practice the intervention at home for at least 30 min and record the starting and ending times in the practice log, the participants often forgot to do so, which made accurate assessment of their compliance levels difficult. At this point, more rigorous and incentive study design is needed in future study. In addition, owing to cost considerations, more accurate polysomography was not used for the measurement of sleep conditions at this time. Furthermore, the relatively small sample size and limited recruitment pool (a single district public health center) may have affected the generalizability of the findings. Finally, due to the research funding and time constraints, a quasi-experiment study was conducted in this study, it may be indispensable to design more rigorous randomized controlled trial, in order to improve the sleep quality of menopausal women in the future.

## 5. Conclusions

In conclusion, the results of this study found that the PMRS and MMRS are both effective multicomponent interventions that can improve the sleep quality of menopausal women. Nevertheless, the effectiveness of the MMRS was superior to that of the PMRS in improving the subjective sleep quality for menopausal women in this study. Additionally, the objective sleep indices indicated that both interventions were effective in decreasing sleep latency, but only the MMRS was effective in reducing total nocturnal awake time. Thus, when caring for menopausal women with a sleep disorder, nurses should consider adopting both the MMRS and PMRS as viable multi-components interventions that are “drug-free,” convenient, safe, and economical ways to ease their sleep problems.

## Figures and Tables

**Table 1:** The descriptions of interventions.

Groups	MMRS (n = 39)	PMRS (n = 39)	Control (n = 39)
Frequency of sessions	2 h per week * four sessions	2 h per week * four sessions	None
Key content of sessions	First session: 1. Instructed on issues relating to the sleep problems of menopausal women. 2. Instructed on topics relating to sleep hygiene, and given a brochure to participants. 3. Shared and discussed sleep problems of participants. 4. Provided practice log and explained how to record the duration and frequency of their practice at home. Second session: 1. Instructed and demonstrated the eight-move exercise’ meditation with diaphragmatic breathing skills 2. Had participants to immediately practice they have learned and encouraged sharing experience. 3. Issued the handouts outlining the contents of the MMR session and asked them to practice at least 30 min every day. Third session: 1. Assessed how the participants had learned in the previous teaching session. 2. Encouraged participants to practice what they had learned with a positive reinforcement. Fourth session: 1. Assessed the helpfulness of what they had learned in that particular session, and their thoughts. 2. Practiced the MMR and answered participants’ problems. 3. Discussed participants’ experiences.4. Emphasized the importance of daily practice and log keeping.	First session: 1. Instructed on issues relating to the sleep problems of menopausal women. 2. Instructed on topics relating to sleep hygiene and gave a brochure to participants. 3. Shared and discussed sleep problems of participants. 4. Provided practice log and explained how to record the duration and frequency of their practice at home. Second session: 1. Explained and demonstrated the procedures of the PMR 2. Asked participants to immediately returned demonstration. 3. Shared and discussed participants’ experiences. 4. Provided CDs with PMR instructions to the participants and asked them to practice at least 30 min every night lying down to fall asleep. Third session: 1. Assessed how the participants had learned the previous teaching session, and encouraged participants to practice what they had learned with a positive reinforcement. 2. Answered and discussed participants’ experiences. Fourth session: 1. Assessed the helpfulness of what participants had learned in that particular session, and their thoughts. 2. Practiced the PMR and answered problems. 3. Discussed participants’ experiences 4. Emphasized the importance of daily practice and log keeping.	1. Received no intervention. 2. Provided the same sleep-promoting brochure given to the experimental groups at the end of intervention.

Note: PMRS: progressive muscle relaxation and sleep hygiene; MMRS: meditative movement relaxation and sleep hygiene. * Duration: four weeks.

**Table 2:** Demographic characteristics at baseline.

Characteristic	Total (n = 117)	PMRS (n = 39)	MMRS (n = 39)	Control (n = 39)	F/χ^2^/Fisher’s Exact
Age, mean (SD)	52.74 (3.9)	53.8 (4.3)	51.8 (3.4)	52.6 (4.09)	2.55
Menopausal status, n (%)					7.43
Pre-menopause	32 (27.1)	6 (15.4)	10 (25.0)	16 (41.0)	
Peri-menopause	21 (17.8)	7 (17.9)	9 (22.5)	5 (12.8)	
Post-menopause	64 (55.1)	26 (66.7)	20 (52.5)	18 (46.2)	
Employment, n (%)					33.47 ***
No	50 (42.4)	28 (71.8)	19 (48.6)	3 (7.7)	
Yes	67 (57.6)	11 (28.2)	20 (51.4)	36 (92.3)	
Hypnotic use, n (%)					0.96
No	89 (75.4)	27 (69.2)	27 (69.3)	35 (89.7)	
Yes	28 (24.6)	12 (30.8)	12 (30.7)	4 (10.3)	
Hormone replacement use, n (%)					4.09
No	111 (94.9)	37 (94.9)	35 (89.6)	39 (100)	
Yes	6 (5.1)	2 (5.1)	4 (10.4)	0 (0)	
Education, n (%)					28.37 **
Elementary	23 (20.3)	15 (38.5)	6 (17.5)	2 (5.1)	
Junior or senior high school	49 (41.5)	19 (48.7)	19 (47.5)	11 (28.2)	
college or above	45 (38.2)	5 (12.8)	14 (35.0)	26 (66.7)	
Marital status, n (%)					7.72
Married	91 (78.0)	32 (82.1)	31 (79.4)	28 (71.8)	
Divorced/widowed	18 (15.3)	7 (17.9)	6 (15.4)	5 (12.8)	
Single	8 (6.7)	0 (0)	2 (5.2)	6 (15.4)	
Exercise, n (%)					2.89
No	68 (58.5)	20 (51.3)	21 (53.8)	27 (69.2)	
Yes	49 (41.5)	19 (48.7)	18 (46.2)	12 (30.8)	
Chronic illnesses, n (%)					8.54
No	70 (60.2)	21 (53.8)	20 (51.4)	29 (74.4)	
Yes	47 (39.8)	18 (38.3)	19 (48.6)	10 (25.6)	
Subjective sleep quality, (mean, SD)	10.81 (.28)	11.4 (56)	11.0 ± 0.47	10.03 ± 0.38	2.23
Objective sleep parameter Onset Latency (min)	20.19 (18.6)	21.03 (22.4)	23.74 (19.1)	15.80 (12.1)	1.87
Snooze (min)	15.23 (16.4)	15.36 (16.7)	14.03 (17.5)	16.29 (15.2)	0.18
Sleep efficiency (%)	80 (8.1)	81.05 (8.1)	81.20 (7.6)	78.07 (8.2)	1.90
WASO (%)	13.49 (6.59)	11.75 (6.3)	12.29 (5.6)	16.42 (6.9)	6.42 **
TST (h)	6.27 (1.1)	5.94 (1.1)	6.55 (0.99)	6.320 (1.2)	2.23

Note: ** *p* < 0.01; *** *p* < 0.001; SD: standard deviation; PMRS: progressive muscle relaxation and sleep hygiene; MMRS: meditative movement relaxation and sleep hygiene; WASO: wake-time after sleep onset; TST: total sleep time.

**Table 3:** Distribution of subjective sleep parameters scores.

Variables	PMRS (n = 39)				MMRS (n = 39)				Control (n = 39)			
	Pre (Mean ± SD)	Post (Mean ± SD)	8-Weeks F/U (Mean ± SD)	12-Weeks F/U(Mean ± SD)	Pre (Mean ± SD)	Post (Mean ± SD)	8-Weeks F/U (Mean ± SD)	12-Weeks F/U (Mean ± SD)	Pre (Mean ± SD)	Post (Mean ± SD)	8-Weeks F/U (Mean ± SD)	12-Weeks F/U (Mean ± SD)
Subjective sleep quality	2.0 ± 0.82	1.36 ± 0.67	1.38 ± 0.71	1.33 ± 0.74	1.93 ± 0.73	1.28 ± 0.60	1.23 ± 0.71	0.90 ± 0.60	1.56 ± 0.85	1.59 ± 0.72	1.36 ± 0.78	1.54 ± 0.72
Sleep latency	41.41 ± 31.64	34.49 ± 21.97	35.13 ± 29.03	25.18 ± 12.80	42.87 ± 30.25	30.51 ± 27.62	28.85 ± 17.82	27.31 ± 17.62	23.13 ± 16.44	24.56 ± 19.77	22.62 ± 17.16	21.72 ± 20.60
Sleep duration (h)	5.41 ± 1.27	5.60 ± 1.24	5.78 ± 1.33	5.95 ± 1.09	5.73 ± 0.74	6.18 ± 0.94	6.63 ± 0.94	6.59 ± 0.95	6.05 ± 0.96	5.78 ± 1.07	6.00 ± 1.25	5.99 ± 1.06
Habitual sleep efficiency (%)	73.80 ± 14.62	79.91 ± 13.06	78.27 ± 14.86	82.55 ± 11.11	77.87 ± 11.67	81.47 ± 11.60	82.16 ± 10.83	82.96 ± 10.31	81.15 ± 12.07	79.72 ± 14.39	80.05 ± 15.32	77.87 ± 17.54
Sleep disturbance	1.51 ± 0.56	1.41 ± 0.50	1.38 ± 0.71	1.31 ± 0.61	1.50 ± 0.56	1.31 ± 0.47	1.26 ± 0.50	1.10 ± 0.45	1.36 ± 0.54	1.31 ± 0.52	1.28 ± 0.56	1.21 ± 0.47
Use of sleeping pills	1.15 ± 1.24	0.74 ± 1.11	0.92 ± 1.18	0.79 ± 1.08	1.05 ± 1.26	0.33 ± 0.86	0.51 ± 0.85	0.44 ± 0.79	0.31 ± 0.69	0.31 ± 0.66	0.23 ± 0.63	0.23 ± 0.48
Daytime dysfunction	1.38 ± 0.63	1.00 ± 0.83	0.97 ± 0.81	0.77 ± 0.67	1.13 ± 0.72	0.69 ± 0.69	0.67 ± 0.53	0.38 ± 0.54	0.74 ± 0.59	0.64 ± 0.63	0.67 ± 0.66	0.64 ± 0.62
Global sleep quality	11.41 ± 3.48	8.56 ± 3.42	8.41 ± 3.60	7.41 ± 3.46	10.95 ± 2.92	7.28 ± 2.71	6.41 ± 3.03	6.67 ± 2.91	7.67 ± 3.18	8.43 ± 3.21	7.90 ± 3.16	8.10 ± 2.79

Note: Plus—minus values are mean *±* SD.

**Table 4:** Distribution of objective sleep parameters scores between pre- and post-intervention.

Variables	PMRS (n = 39)		MMRS (n = 39)		Control (n = 39)	
	Pre	Post	Pre	Post	Pre	Post
	Mean ± SD	Mean ± SD	Mean ± SD	Mean ± SD	Mean ± SD	Mean ± SD
Onset Latency (min)	21.03 ± 22.43	14.37 ± 12.08	23.74 ± 19.16	15.81 ± 12.61	15.80 ± 12.12	16.79 ± 13.33
Sleep efficiency (%)	81.05 ± 8.13	82.37 ± 6.66	81.20 ± 7.65	84.15 ± 7.57	78.07 ± 8.21	77.36 ± 10.13
WASO (%)	13.75 ± 6.30	10.01 ± 3.71	12.29 ± 5.64	9.72 ± 5.06	12.42 ± 6.90	14.05 ± 9.36
Snooze (min)	15.36 ± 14.75	13.24 ± 12.39	14.03 ± 17.57	9.88 ± 9.39	16.29 ± 15.16	9.62 ± 8.12
TST	5.94 ± 1.05	6.12 ± 1.08	6.55 ± 0.99	6.42 ± 1.34	6.32 ± 1.17	5.89 ± 1.08
TST (%)	88.33 ± 6.38	87.55 ± 7.83	87.71 ± 5.64	88.33 ± 7.14	83.35 ± 7.27	82.03 ± 9.36

Note: WASO: wake-time after sleep onset; TST: total sleep time.

**Table 5:** The effects of intervention on subjective and objective sleep parameters of participants.

Parameter	Β (Estimate)	S E	Wald *x*^2^	*p*-Value
Global PSQI				
Intercept	8.54	1.02	69.67	<0.001
Group				
PMRS vs. control	3.23	0.91	12.38	<0.001
MMRS vs. control	3.20	0.76	17.46	<0.001
Time				
T2 vs. T1	0.18	0.46	0.15	0.69
T3 vs. T1	−0.54	0.49	1.19	0.28
T4 vs. T1	−0.15	0.41	0.14	0.71
Group × Time				
Group(PMRS) × T 2 ^†^	−2.51	0.67	14.03	<0.001
Group(PMRS) × T3 ^†^	−1.69	0.71	5.61	0.02
Group(PMRS) × T4 ^†^	−3.05	0.67	20.72	<0.001
Group(MMRS) × T2 ^†^	−3.97	0.68	34.21	<0.001
Group(MMRS) × T3 ^†^	−3.54	0.70	25.92	<0.001
Group(MMRS) × T4 ^†^	−4.92	0.64	59.79	<0.001
Group(MMRS) × T2 ^††^	−1.46	0.70	4.40	0.04
Group(MMRS) × T3 ^††^	−1.85	0.71	6.75	0.01
Group(MMRS) × T4 ^††^	−1.92	0.72	7.11	0.01
Education level ^§^				
Junior or senior high school	−0.45	0.83	0.29	0.59
College or above	−0.95	0.86	1.34	0.28
Employment status *				
No	−0.1	0.66	0.05	0.83
Actigraphic parameters				
1. Sleep Onset Latency				
Intercep	11.62	3.22	13.02	<0.001
Group				
PMRS vs. control	7.23	4.06	3.17	0.15
MMRS vs. control	8.93	3.49	6.56	0.03
Time				
T2 vs. T1	0.99	1.96	0.26	0.61
Group × Time				
PMRS × T2 ^†^	−7.66	3.67	4.36	0.04
MMRS × T2 ^†^	−8.93	3.63	6.05	0.01
MMRS × T2 ^††^	−1.27	4.35	0.09	0.77
Education level ^§^				
Junior or senior high school	3.20	3.32	0.93	0.34
College or above	4.93	3.08	2.56	0.11
Employment status *				
No	−3.50	2.70	1.69	0.20
2. Wake time (%)				
Intercep	14.93	1.61	95.52	<0.001
Group				
PMRS vs. control	−3.95	1.59	7.96	0.01
MMRS vs. control	−3.74	1.47	7.72	0.01
Time				
T2 vs. T1	4.45	7.09	0.39	0.53
Group × Time				
PMRS × T2 ^†^	−3.36	1.85	3.29	0.07
MMRS × T2 ^†^	−4.20	1.78	5.55	0.02
MMRS × T2 ^††^	−0.83	1.36	0.37	0.54
Education level ^§^				
Junior or senior high school	0.86	1.14	0.57	0.45
College or above	0.68	1.31	0.27	0.61
Employment status *				
No	1.08	0.94	1.34	0.25
3. Sleep Efficiency				
Intercep	81.50	2.16	1791.39	<0.001
Group				
PMRS vs. control	2.11	1.96	1.16	0.28
MMRS vs. control	2.80	1.83	2.35	0.13
Time				
T2 vs. T1 ^§^	−0.70	1.64	0.18	0.67
Group × Time				
PMRS × T2 ^†^	2.02	2.11	0.92	0.34
MMRS × T2 ^†^	3.64	2.06	3.14	0.08
MMRS × T2 ^††^	1.62	1.80	0.81	0.37
Education level ^§^				
Junior or senior high school	−2.67	1.51	3.12	0.08
College or above	−2.65	1.63	2.65	0.10
Employment status *				
No	−1.21	1.43	0.71	0.40
4. Snooze Time				
Intercep	11.70	3.81	9.42	<0.01
Group				
PMRS vs. control	−0.51	3.77	0.02	0.91
MMRS vs. control	−1.71	3.65	0.44	0.64
Time	−6.68	2.56	6.79	<0.01
T2 vs. T1 ^§^				
Group × Time				
PMR × T2 ^†^	4.56	3.62	1.58	0.21
MMRS × T2 ^†^	2.53	4.06	0.388	0.53
MMRS × T2 ^††^	−2.03	4.06	0.25	0.62
Education level ^§^				
Junior or senior high school	4.74	2.77	2.93	0.09
College or above	2.61	2.46	1.12	0.29
Employment status *				
No	2.06	2.99	0.48	0.49
5. Total sleep time (min)				
Intercep	395.62	19.27	421.34	<0.001
Group				
PMRS vs. control	−34.76	16.58	4.40	0.04
MMRS vs. control	6.46	16.06	0.16	0.69
Time				
T2 vs. T1 ^§^	−25.82	11.70	5.48	0.02
Group × Time				
PMRS × T2 ^†^	36.66	16.07	5.21	0.02
MMRS × T2 ^†^	26.38	16.55	2.53	0.11
MMRS × T2 ^††^	−10.28	16.55	0.39	0.53
Education level ^§^	−0.45	1.48	0.09	0.76
Junior or senior high school				
College or above	−0.13	1.59	0.01	0.93
Employment status *	−11.95	11.24	1.13	0.29
No				

Note: T1: before intervention; T2: post-intervention; T3: 8 week follow-up; T4: 12 week follow-up; ^§^ reference group: elementary or illiterate; * reference group: in employment; ^†^ reference group: control group × T1; ^††^ reference group: PMRS group × T1.

## Data Availability

Not applicable.
